# Basilar artery dolichoectasia presenting as obstructive hydrocephalus mimicking normal pressure hydrocephalus

**DOI:** 10.1093/omcr/omag076

**Published:** 2026-05-24

**Authors:** Jarrel Watson, Vijai Persaud, Asif Subhan

**Affiliations:** Neurology Department, Georgetown Public Hospital Corporation, New Market Street, North Cummingsburg, Georgetown, Demerara-Mahaica Region, Guyana; Department of Radiology (Neuroradiology), Georgetown Public Hospital Corporation, New Market Street, North Cummingsburg, Georgetown, Demerara-Mahaica Region, Guyana; Department of Neurosurgery, Georgetown Public Hospital Corporation, New Market Street, North Cummingsburg, Georgetown, Demerara-Mahaica Region, Guyana

**Keywords:** basilar artery dolichoectasia, obstructive hydrocephalus, normal pressure hydrocephalus mimic, vertebrobasilar dolichoectasia, ventriculoperitoneal shunt

## Abstract

Vertebrobasilar dolichoectasia is a well-recognized vascular anomaly characterized by tortuosity, elongation, and dilatation of the vertebrobasilar arteries with associated hemodynamic changes. Hydrocephalus secondary to this condition is rare but may clinically mimic idiopathic normal pressure hydrocephalus (iNPH). We report the case of a 58-year-old man with poorly controlled hypertension who presented with progressive gait instability, cognitive impairment, and urinary incontinence over one month. Neuroimaging demonstrated ventriculomegaly with transependymal oedema and marked vertebrobasilar dolichoectasia, with the basilar artery compressing the floor of the third ventricle. Quantitative imaging supported a diagnosis of obstructive hydrocephalus rather than iNPH. This case highlights an important vascular cause of secondary hydrocephalus and emphasizes the need for careful radiological evaluation in patients presenting with the classical triad of normal pressure hydrocephalus.

## Introduction

Vertebrobasilar dolichoectasia is defined by elongation, dilatation, and tortuosity of the vertebrobasilar arterial system [1–3]. The diagnostic criteria for VBD are arterial diameter of over 4.5 mm at anywhere along its course and deviation of any portion of vessel higher than 10 mm from the shortest expected course, basilar length of over 29.5 mm, or intracranial vertebral artery length of over 23.5 mm [1]. The condition is associated with long-standing hypertension, male sex, and advancing age, and may present clinically with ischemic stroke, intracranial hemorrhage, or compressive brainstem syndrome [2–5].

Hydrocephalus resulting from vertebrobasilar dolichoectasia is uncommon but represents an important and potentially reversible cause of secondary hydrocephalus [6, 7].

Recognizing this rare mechanism is clinically important, as patients presenting with the classical triad of normal pressure hydrocephalus may instead have a secondary and potentially treatable cause of ventricular enlargement.

## Case report

A 58-year-old right-handed man with poorly controlled hypertension presented with a one-month history of progressive gait instability, short-term memory impairment, and urinary incontinence. He also reported an intermittent bifrontal headache and an episode of non-projectile vomiting. There was no history of fever, seizures, head trauma, or recent infection. The patient was a chronic smoker who had regularly consumed alcohol.

During examination, he remained alert yet intermittently disoriented, exhibiting variable attention and reduced short-term memory. Muscle strength measured 4/5 on the Medical Research Council scale across all muscle groups. There was generalized hyperreflexia without any abnormal reflexes. Cerebellar assessment revealed upper limb dysmetria and difficulty with rapid alternating movements. His gait was broad-based and unsteady, necessitating support. Additionally, the patient experienced urinary incontinence.

### Neuroimaging

**Figure 1:**
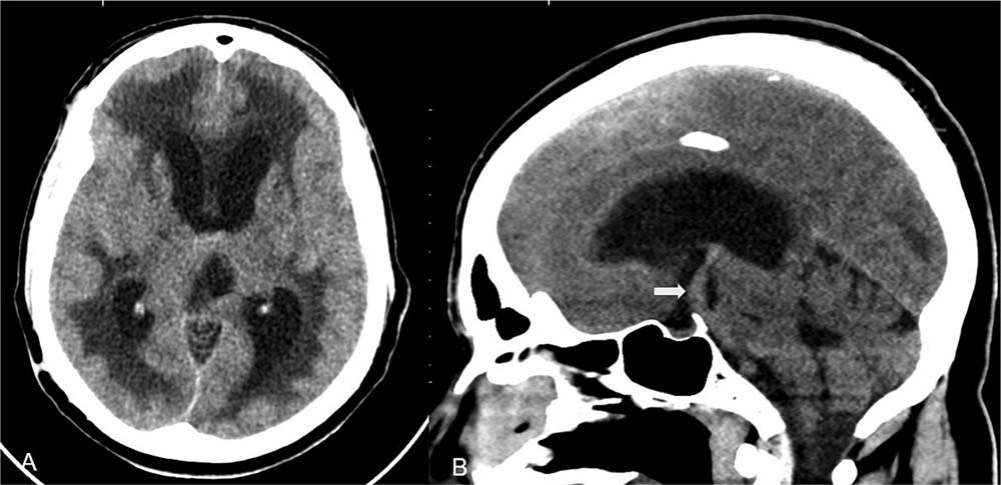
Non-contrast CT of the brain at presentation. (A) Axial CT image at the level of the lateral ventricles demonstrating ventriculomegaly with periventricular hypodensity consistent with transependymal cerebrospinal fluid (CSF) permeation. (B) Mid-sagittal CT reconstruction showing an elongated and ectatic basilar artery indenting the floor of the third ventricle.

Non-contrast computed tomography (CT) of the brain demonstrated enlargement of the lateral and third ventricles with relative sparing of the fourth ventricle, consistent with obstructive hydrocephalus. (Fig. 1) Periventricular hypodensity indicates transependymal cerebrospinal fluid (CSF) permeation.

**Figure 2:**
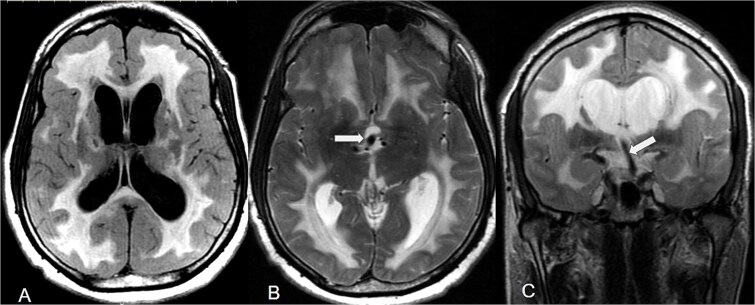
MRI of the brain demonstrating obstructive hydrocephalus secondary to vertebrobasilar dolichoectasia. (A) Axial FLAIR image and (B) axial T2-weighted image demonstrating dilatation of the lateral and third ventricles with periventricular hyperintensity consistent with transependymal oedema. (C) Coronal T2-weighted image demonstrating an ectatic basilar artery compressing the floor of the third ventricle.

Magnetic resonance imaging (MRI) confirmed ventriculomegaly with periventricular T2 and fluid attenuated inversion recovery (FLAIR) hyperintensity consistent with transependymal oedema. (Fig. 2).

Basilar artery measurements aligned with vertebrobasilar dolichoectasia per Smoker’s criteria. The basilar artery diameter was approximately 6 mm, surpassing the typical threshold of > 4.5 mm. Coronal imaging showed a reduced callosal angle. The basilar artery was elongated, with upward displacement of the bifurcation and indentation of the third ventricle floor. The vertebral arteries did not show significant ectasia. Vesicloventricular enlargement was evident, affecting the lateral and third ventricles, with relative sparing of the fourth ventricle. The Evans index was elevated (>0.3), indicating hydrocephalus rather than age-related ventricular enlargement.

Chronic infarcts were observed on both sides of the corona radiata. Diffusion-weighted imaging showed an acute infarct in the right centrum semiovale. No intracranial hemorrhage or mass lesion was detected.

### Outcome and follow-up

**Figure 3:**
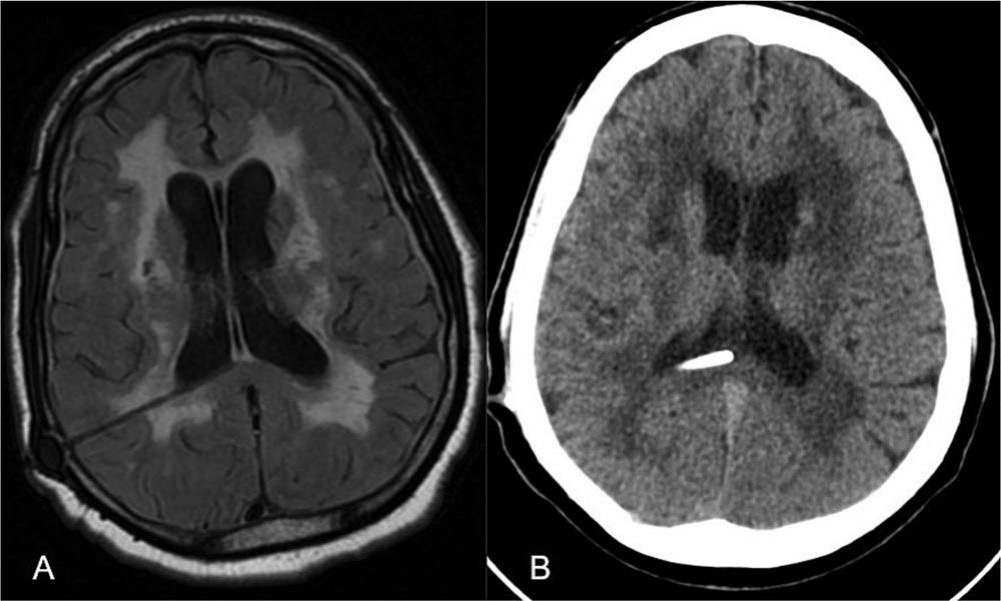
Post-operative imaging following ventriculoperitoneal shunt placement. (A) Axial T2-FLAIR MRI at 2 months demonstrating reduction in transependymal oedema. (B) Non-contrast CT at 6 months demonstrating decreased ventriculomegaly with ventriculoperitoneal shunt catheter in situ.

At the two-month follow-up after ventriculoperitoneal shunt placement, the patient showed significant improvements in gait stability and cognitive function, with the resolution of urinary incontinence. Follow-up imaging showed a reduction in ventricular size and decreased transependymal edema (Fig. 3).

## Discussion

Although vertebrobasilar dolichoectasia is most commonly associated with ischemic stroke or brainstem compression, hydrocephalus represents a recognized but uncommon complication [6, 7].

Secondary hydrocephalus in vertebrobasilar dolichoectasia may occur through direct mechanical obstruction of cerebrospinal fluid (CSF) pathways or disturbance of normal CSF pulsatile dynamics [6, 8]. In the present case, the markedly elongated and ectatic basilar artery indented the floor of the third ventricle, producing functional obstruction at the level of the third ventricular outflow and proximal aqueduct. This resulted in enlargement of the lateral and third ventricles with relative preservation of the fourth ventricle, a pattern consistent with obstructive hydrocephalus. This pattern has been described in prior reports of vascular compression causing ventricular outflow obstruction (10). In addition to static mechanical compression, pronounced arterial pulsatility in dolichoectatic vessels may further impair CSF circulation. This mechanism has been described as ‘obstructive-invisible hydrocephalus,’ in which countercurrent pulsations of the basilar artery extend into the floor of the third ventricle and generate a water-hammer–like pulse transmitted toward the foramina of Monro, thereby impairing CSF outflow from the lateral ventricles [8].

The imaging findings in this patient, particularly ventricular enlargement involving the lateral and third ventricles with relative sparing of the fourth ventricle and direct indentation of the third ventricular floor by the ectatic basilar artery, support this pathophysiological mechanism.

An important diagnostic consideration in this case was idiopathic normal-pressure hydrocephalus (iNPH), given the classical clinical triad of gait disturbance, cognitive decline, and urinary incontinence. However, radiological features including transependymal oedema, disproportionate enlargement of the lateral and third ventricles, and direct vascular compression supported an obstructive aetiology rather than a communicating process [3, 6]. These findings highlight the importance of careful evaluation of structural causes before attributing the clinical syndrome to idiopathic normal pressure hydrocephalus.

There are currently no established treatment guidelines for hydrocephalus secondary to vertebrobasilar dolichoectasia [7]. Ventriculoperitoneal shunting remains the most commonly reported intervention and may provide symptomatic improvement when hydrocephalus is clinically significant [7].

Endoscopic third ventriculostomy has also been described as a potential treatment option in selected cases [9]. However, in the presence of a markedly ectatic basilar artery closely approximating the floor of the third ventricle, the procedure may carry an increased risk of vascular injury. For this reason, ventriculoperitoneal shunting was considered the safer and more appropriate intervention in this patient.

Recognition of vertebrobasilar dolichoectasia as the underlying etiology has important diagnostic and clinical implications [7, 10]. Unlike idiopathic normal pressure hydrocephalus, this condition reflects an underlying vascular pathology that may predispose patients to additional complications, including ischemic stroke, brainstem compression, and cranial nerve dysfunction [4, 7]. Although ventriculoperitoneal shunting may also be performed in idiopathic normal pressure hydrocephalus, identifying a secondary obstructive cause is important because it may influence surgical planning, prognosis, and long-term surveillance for complications related to vertebrobasilar dolichoectasia, including progressive vascular compression and cerebrovascular events.
